# How to use learning curves to evaluate the sample size for malaria prediction models developed using machine learning algorithms

**DOI:** 10.1186/s12936-025-05479-3

**Published:** 2025-07-24

**Authors:** Sophie G. Zaloumis, Megha Rajasekhar, Julie A. Simpson

**Affiliations:** 1 Centre for Epidemiology and Biostatistics, Melbourne School of Population and Global Health, University of Melbourne, Carlton, VIC Australia; 2 MISCH (Methods and Implementation Support for Clinical Health) Research Hub, Faculty of Medicine, Dentistry, and Health Sciences, University of Melbourne, Carlton, VIC Australia; 3 Nuffield Department of Medicine, University of Oxford, Oxford, UK

**Keywords:** Machine learning, Sample size, Prediction modelling, Learning curves, Malaria, Transcriptomics

## Abstract

**Background:**

Machine learning algorithms have been used to predict malaria risk and severity, identify immunity biomarkers for malaria vaccine candidates, and determine molecular biomarkers of antimalarial drug resistance. Developing these prediction models requires large training datasets to ensure prediction accuracy when applied to new individuals in the target population. Learning curves can be used to assess the sample size required for the training dataset by evaluating the predictive performance of a model trained using different dataset sizes. These curves are agnostic to the specific prediction model, but their construction does require existing data. This tutorial demonstrates how to generate and interpret learning curves for malaria prediction models developed using machine learning algorithms.

**Methods:**

To illustrate the approach, training dataset sizes were evaluated to inform the design of a “mock” prediction modelling study aimed to predict the artemisinin resistance status of *Plasmodium falciparum* malaria isolates from gene expression data. Data were simulated based on a previously published in vivo parasite gene expression dataset, which contained transcriptomes of 1043 *P. falciparum* isolates from patients with acute malaria, of which 29% (299/1043) were from slow clearing infections (parasite clearance half-life > 5 h). Learning curves were produced for two machine learning algorithms, sparse Partial Least Squares-Discriminant Analysis plus Support Vector Machines (sPLSDA + SVMs) and random forests. Prediction error was measured using the balanced error rate (average of percentage of slow clearing infections incorrectly predicted as fast and percentage of fast clearing infections predicted as slow).

**Results:**

For this mock malaria prediction study, the balanced error rate on a test dataset not used for model training (208 samples) was 50% for sPLSDA + SVMs and 50% for random forests on the smallest training dataset evaluated (20 samples) and 14% for sPLSDA + SVMs and 22% for random forests on the largest training dataset evaluated (835 samples). The shape of the learning curves indicates that increasing the training dataset size beyond 835 samples is unlikely to significantly reduce the balanced error rates further.

**Conclusions:**

Learning curves are a simple tool that can be used to determine the minimum sample size required for future prediction modelling studies of different malaria outcomes that use machine learning algorithms for prediction. These curves need to be generated for each specific prediction modelling application.

**Supplementary Information:**

The online version contains supplementary material available at 10.1186/s12936-025-05479-3.

## Background

Machine learning algorithms are becoming widely adopted in malaria prediction modelling studies where the aims range from predicting malaria risk and severity, identifying immunity biomarkers for malaria vaccine candidates, and detecting molecular biomarkers of antimalarial drug resistance. To illustrate this, a cursory search of Web of Science (all databases) identified 123 journal articles that included the terms malaria, machine learning and prediction in their title, abstract and/or keywords over the last 5 years (2019–2023) and increased from 10 in 2019 to 36 in 2023. It is common for no sample size calculations or assessments to be performed a priori in prediction modelling studies, and as Riley et al*.* [1, 2] highlight, in clinical research more broadly, many prediction models are developed using datasets (commonly referred to as the training dataset) that are too small for the number of participants and outcome events. A priori consideration of how sensitive model predictions are to variation in training dataset size and composition, could improve the reliability of prediction models when applied to new individuals in the target population.

Most clinical prediction models are developed using conventional statistical regression models, such as linear or logistic regression. Up until recently, sample size determination for the training dataset has been ad hoc for regression-based prediction modelling. For example, a simple rule of thumb of 10 events (e.g. malaria cases) per predictor variable (i.e., explanatory variable) is typically used for logistic regression [3]. Riley et al*.* [1, 4, 5] published recommendations for calculating the sample size needed to develop a clinical prediction model, and present a procedure for continuous, binary and survival (time-to-event) outcomes, where the aim is to minimise the potential for model overfitting and to estimate key parameters evaluating the performance of the prediction model precisely (e.g. the overall outcome proportion and predicted outcome probabilities for new individuals in the case of binary outcomes).

Machine learning algorithms, such as random forests and neural networks, are an alternative to regression-based prediction models [6, 7]. Machine learning algorithms are typically adopted when the number of predictors is greater than the number of observations, for example, malaria studies that have measured antibody features [8] and host transcriptional signatures to distinguish falciparum malaria infection from bacterial infection and cerebral malaria from a different clinical phenotype (e.g., uncomplicated malaria and severe malarial anaemia) [9]. Due to the complexity of machine learning algorithms (e.g., a deep convolutional neural network with millions of parameters), traditional sample size calculations cannot be applied or are not straightforward for prediction models developed using these methods [10]. An approach to assessing the training dataset sample size required to develop a prediction model is a learning curve, which evaluates the prediction error of a model at different training dataset sample sizes [11–15]. Silvey and Liu [15] showed that the sample size required for prediction models of a binary outcome depends on the content area of investigation and the machine learning algorithm.

The aim of this tutorial is to illustrate how to generate and interpret a learning curve to inform the study design of a “mock” prediction modelling study aiming to predict the artemisinin resistance status of *Plasmodium falciparum* malaria isolates using in vivo transcription data. To illustrate the concepts behind learning curves, a toy scenario for a continuous outcome and a continuous predictor will be described in sections “Learning curves: construction” and “Learning curves: interpretation”. How these concepts apply to the classification setting where the outcome is binary or categorical is discussed in the section “Classification problems”. The remainder of this tutorial provides an example of how learning curves can be used to inform the design of the mock malaria prediction modelling study. The methods described in this tutorial are suitable for supervised learning problems, where for each observation of the predictor measurement(s), there is an associated outcome measurement (e.g. severe *P. falciparum* case).

## Methods

### Learning curves: construction

To generate a learning curve the following information and data needs to be available at the design stage of a study: a proposed prediction model or prediction model development process, and data from either a previous study or simulation containing outcomes and candidate predictors that resemble those that will be obtained from the target population of the study.

The following notation and concepts are required to set up a toy scenario to illustrate the basic ideas behind learning curves. Let $x$ be a continuous predictor (e.g. total IgG levels to an antigen of interest) and $y$ be a continuous outcome variable (e.g. parasitaemia) and say data $\{\left({x}_{i},{y}_{i}\right)\}$ has been collected from $i=1,\dots ,N\left(=100\right)$ individuals. In supervised learning, it is assumed that there exists a true model ($f$) that perfectly describes the relationship between the outcome and the candidate predictors [16]. In practice this model is typically unknown, and we estimate this unknown relationship with our prediction model ($\widehat{f}$) [16].

Some examples of prediction models ($\widehat{f}$ s) for continuous outcome variables that could be selected to estimate the true $f$ are linear regression models and random forests. Lastly, a measure of prediction error, or of how well predictions from $\widehat{f}$ match the observed outcome $y$, needs to be selected. For continuous outcomes, a commonly used measure of prediction error is the mean squared error ($MSE$):

1 $$MSE=\frac{1}{n}{\sum }_{i=1}^{N}{\left({y}_{i}-\widehat{f}\left({x}_{i}\right)\right)}^{2}$$

The $MSE$ will be small if the predictions $\widehat{f}$ are close to the observed outcomes $y$ and will be large if the predicted and observed outcomes differ greatly.

**Fig. 1:**
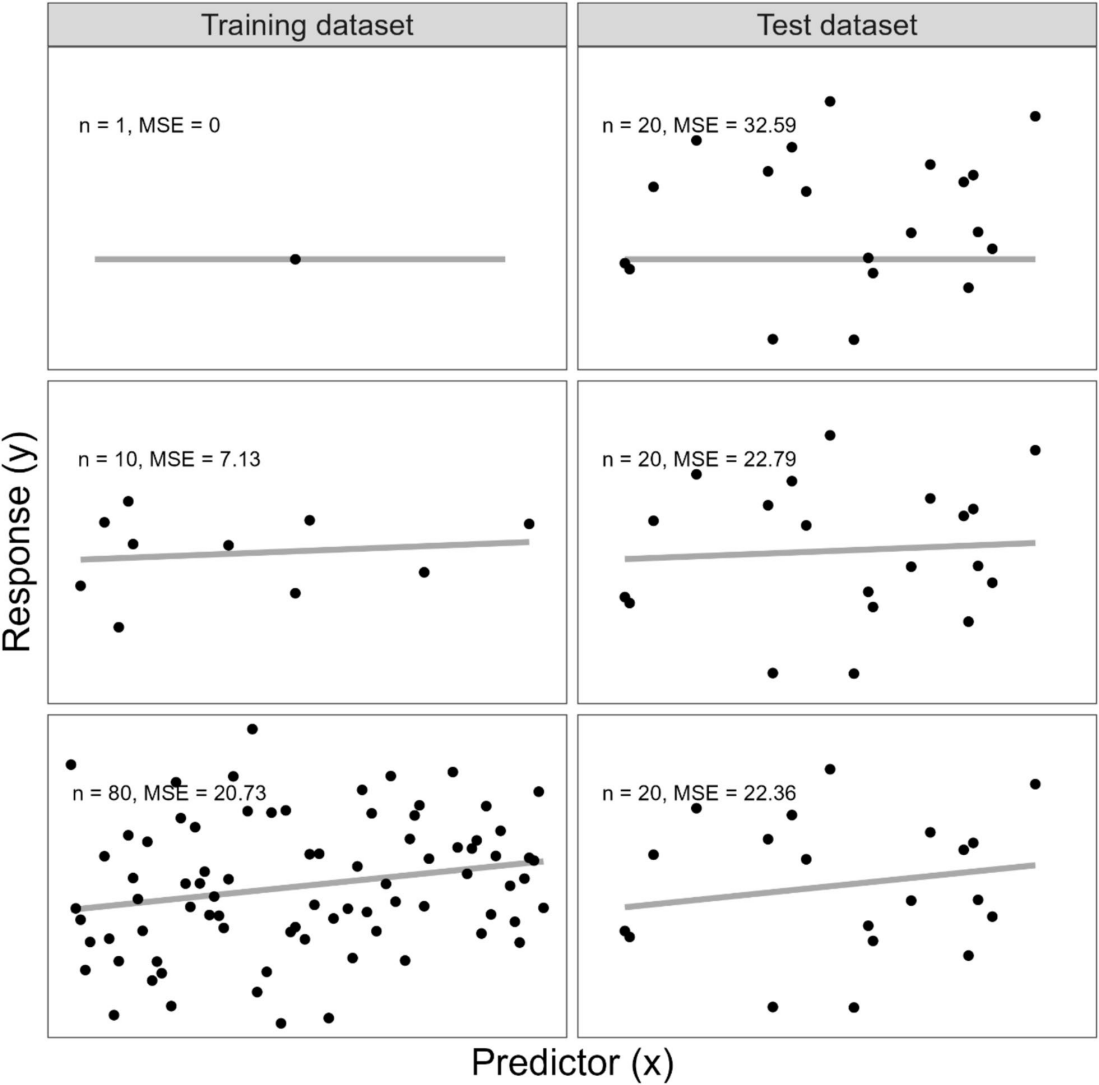
Illustration of how model predictions made on the training datasets and test dataset vary with training dataset size. Black dots represent individuals in the training dataset in left column and test dataset in right column. The grey line represents the fitted model over the range of the predictor variable (x) in the training (i.e., data the model was trained on; left column) and test dataset (i.e., data not involved in model training; right column). Size of training datasets and test dataset provided in top left corner of each panel. Based on a figure from [17]

**Fig. 2:**
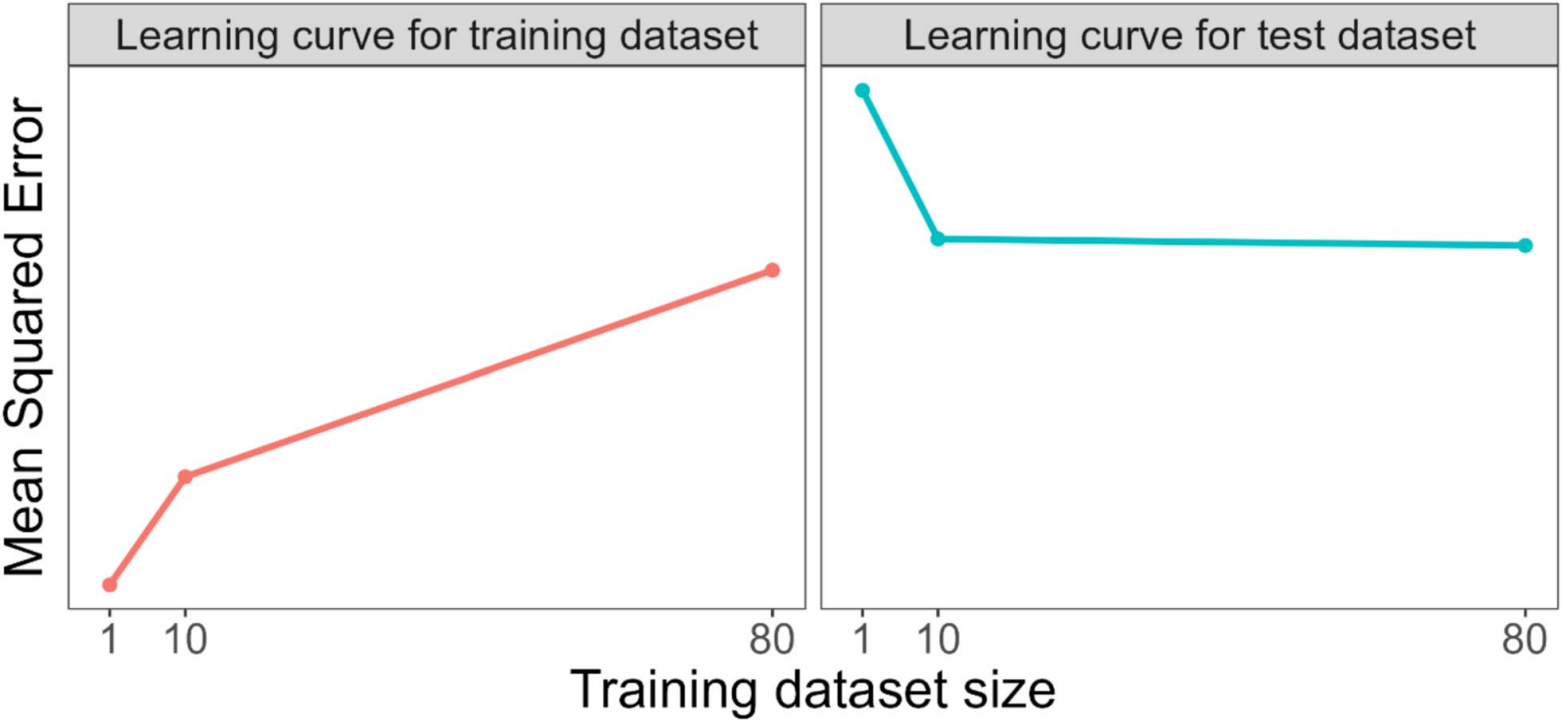
Learning curves derived from training set sizes and mean squared errors for the predictions on the training datasets (n = 1, 10, 80) and test dataset (n = 20) in Fig. 1. The learning curves show how the prediction error changes for the training datasets (left) and test dataset (right), as training dataset size increases

Figure 1 illustrates how learning curves would be constructed for this scenario. For the purposes of the toy example assume a linear regression model is an appropriate choice for the prediction model, $\widehat{f}$. To construct a learning curve, the data are split into a training dataset and test dataset. For example, in Fig. 1 data from 20 individuals are set aside as the test dataset; the prediction model is never trained on (i.e., fit to) the test dataset. Data from the remaining 80 individuals are used to construct training datasets, which the prediction model is trained on (i.e., fit to). In the training dataset column of Fig. 1, the size of the training datasets is increasing, while the size of the test dataset stays the same (i.e. 20 individuals in this example). In the first row, the training dataset size is one individual and the prediction model fits the outcome for that individual perfectly ($MSE$ = 0). However, predictions from that same model fit the test dataset of 20 different individuals poorly, resulting in a much higher $MSE$ on the test dataset. As the training dataset size increases, the prediction model cannot fit the training dataset perfectly anymore and the $MSE$ for the training dataset becomes larger. However, as the model is trained on more data, it starts to fit the test dataset better and the test $MSE$ decreases. Learning curves compare the $MSE$ (or another selected measure of prediction error) for the training datasets and the test dataset, as the training dataset size increases (as shown in Fig. 2 for the toy example).

### Learning curves: interpretation

**Fig. 3:**
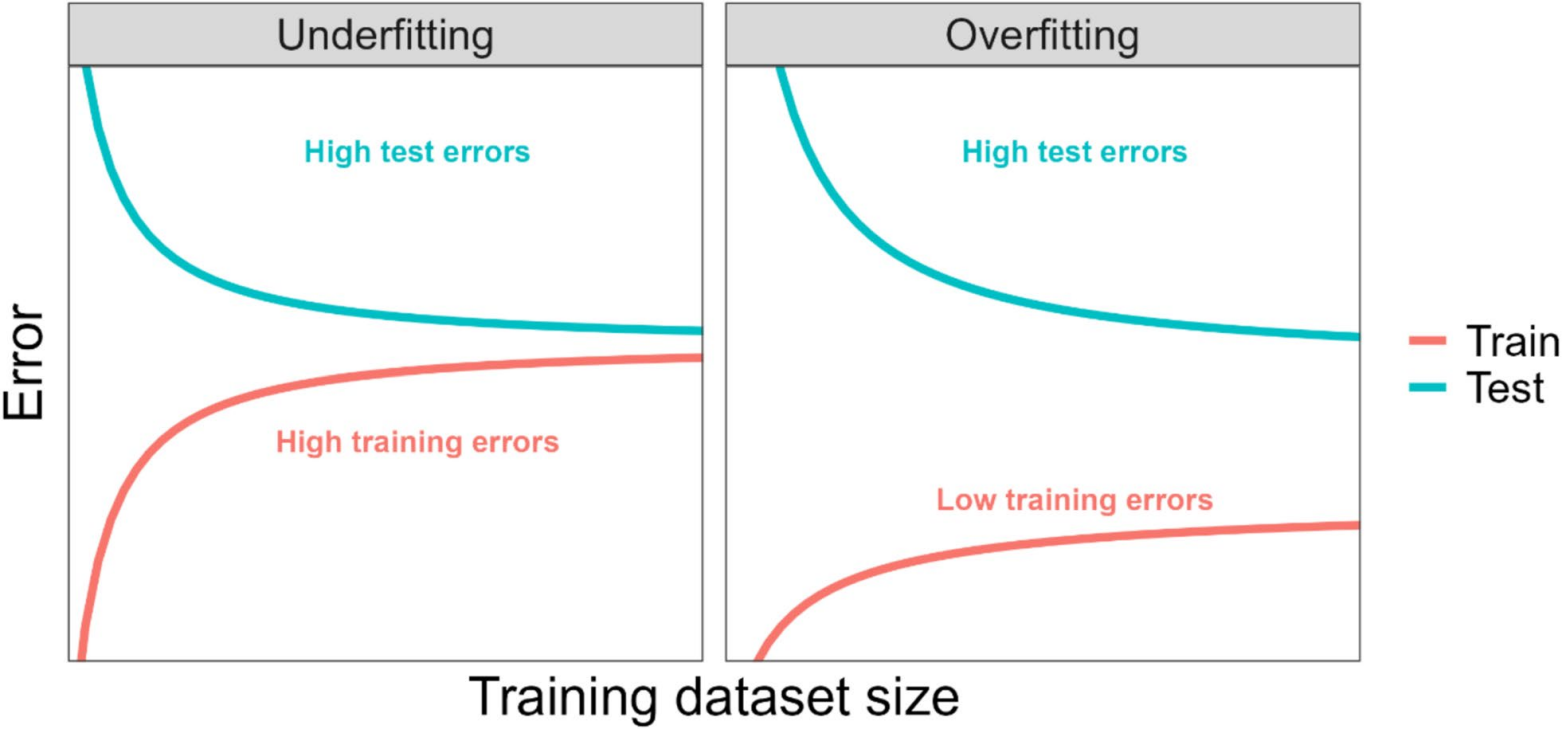
An illustration of how learning curves can be used to examine whether a prediction model is underfitting or overfitting the training dataset. Error represents a suitable measure of prediction error, e.g. mean squared error. Based on a figure from [17]

Learning curves can help us examine whether a prediction model’s poor performance (high error on the test dataset) is due to the model underfitting or overfitting the training data, which, in turn, can help us determine whether increasing the training dataset sample size will improve a prediction model’s accuracy on unseen data. Figure 3 illustrates how learning curves can be used to examine whether a prediction model is underfitting or overfitting the training data (see chapter “Measuring the Quality of Fit” of [16] and chapter “2.9 Model Selection and the Bias-Variance Tradeoff” of [18]).

A high error on the test dataset indicates that the trained model predicts the test data poorly (both left and right panels of Fig. 3). The magnitude/size of the errors on the training dataset can help identify if the high error on the test dataset arose because the model (1) fits the training data poorly because it is too simplistic to capture the relationship between the predictors and outcome (*underfitting* the training data) or (2) fits the training data too well as it captures random fluctuations/noise in the training dataset, rather than the relationship between the predictors and outcome (*overfitting* the training data). In both cases the trained model will have difficulty making accurate predictions on new/unseen data and exhibit high error on the test dataset.

In the scenario where the model does not fit the training data well (i.e., underfitting the training data), the learning curve for the training dataset and test dataset will show high errors as the training dataset size increases and there will be a narrow gap between the training and test curves (left panel of Fig. 3). In the scenario where the model fits the training data too well (i.e., overfitting the training data), the model will exhibit low errors on the training dataset and high errors on the test dataset and there will be a wide gap between the curves as the training dataset size increases (right panel of Fig. 3).

Identifying whether a model is underfitting or overfitting the training data can help us determine whether increasing the training dataset sample size will improve a model’s predictive performance. In the case where the model is underfitting the training data adding more training data is unlikely to help, as the two curves have either converged or appear to be converging to a high error as the training dataset size increases (left panel of Fig. 3).

In the case where the model is overfitting the training data adding more training data is likely to help, i.e., the test curve could converge toward the training curve if more training data were added (right panel of Fig. 3) due to the larger datasets providing more information for the model to learn from and reducing the chance of it modelling noise rather than underlying patterns in the data. This causes the training error to increase slightly and the test error to decrease and the curves to converge towards each other or the gap between the curves to reduce. The ideal scenario is where both the training and test curves have converged to a low error. Note the concept of irreducible error prevents these curves from converging to zero [16, 18].

In the case of underfitting the training data, where adding more training data is unlikely to help, some strategies to overcome this scenario are to change to a more complex model (e.g. random forests or support vector machines (SVM)), include interaction terms between predictors or additional transformations of the predictors to capture nonlinear relationships or consider whether more predictor variables can be collected (i.e., additional predictors included in the model).

### Classification problems

Many of the concepts encountered in the toy example for continuous outcomes, such as underfitting, overfitting and the interpretation of learning curves, apply to the classification setting [16]. As the outcome $y$ is no longer continuous in classification problems, but binary or categorical, the only modification required in the classification setting is to select a measure of prediction error suitable for binary/categorical outcomes [16]. In the classification setting, measures of prediction error are derived from the confusion matrix – a cross tabulation of the observed binary/categorical outcome and the predicted binary/categorical outcome. The elements of a confusion matrix are defined in Table 1 for a binary outcome with categories positive and negative (e.g. malaria case yes/no).

**Table 1:** Elements of the confusion matrix for a binary outcome

	Predicted outcome	
Observed outcome	Positive	Negative
Positive	True positive (TP)	False negative (FN)
Negative	False positive (FP)	True negative (TN)

A common measure of prediction error for classification problems is the balanced error rate (BER), which is the average of the false negative rate (FNR) and false positive rate (FPR) and indicates how likely it is that an individual in a particular category will be classified incorrectly. The FNR (1—sensitivity) is the proportion of observed positive outcomes that are incorrectly predicted by the model and is calculated from the confusion matrix as FN / (TP + FN). FPR (1—specificity) is the proportion of observed negative outcomes that are incorrectly predicted by the model and is calculated from the confusion matrix as FP/(TN + FP). The confusion matrix and balanced error rate can also be derived for outcomes with more than two categories/classes.

### Mock study: motivating data for a malaria prediction model

The remainder of this tutorial aims to demonstrate how to generate and interpret learning curves for malaria prediction models developed using machine learning algorithms. To illustrate the approach, training dataset sizes will be evaluated to inform the design of a “mock” prediction modelling study aimed to predict the artemisinin resistance status of *P. falciparum* malaria isolates utilizing in vivo transcription data.

The motivating data for the mock prediction modelling study is from a large-scale genome-wide association study published by Mok et al*.* [19]. The objective of this study was to characterize the gene expression signatures associated with in vivo artemisinin-resistance. In brief, this study employed microarray technology to determine the global gene expression profiles of isolates sampled from 1043 patients with acute falciparum malaria enrolled into the Tracking Resistance to Artemisinin Collaboration (TRAC) study in 2011–2012 [20]. The samples were taken prior to artemisinin-based combination therapy (ACT) treatment. After treatment with ACT, these patients displayed differential rates of parasite clearance. The samples originated from 14 field sites across South East Asia (Pailin, Pursat, Preah Vihear, Rattanakiri in Cambodia; Mae Sot, Srisakhet, Khun Han, Ranong in Thailand; Shwe Kyin in Myanmar; Binh Phuoc in Vietnam; Attapeu in Laos), Bangladesh and Democratic Republic of Congo. The overall abundance of mRNA transcripts for 5061 of the ~ 5591 genes in the *P. falciparum* genome was measured.

Microarray data and a parasite clearance outcome were simulated based on the TRAC transcriptomics data and parasite clearance half-lives (defined in section “Outcome simulation”) published in Mok et al. [19] and the Supplementary Material of Mok et al*.* [19], respectively.

### Microarray data simulation

Microarray data for 1043 participants/samples were simulated from a multivariate normal distribution with mean vector set to the sample means of the TRAC transcriptomics measurements and covariance matrix set to the sample covariance matrix of the TRAC transcriptomics measurements (Additional file 1).

**Fig. 4:**
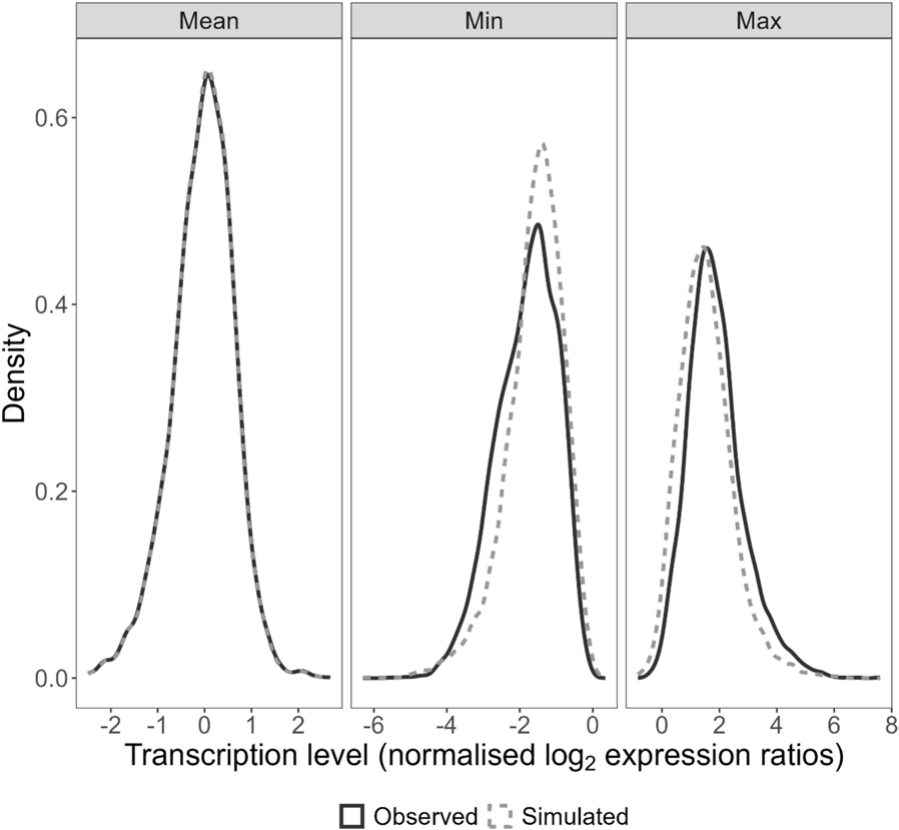
Comparison of observed and simulated transcript abundances. In both the observed TRAC and simulated datasets there are 5061 transcript abundances for 1043 participants/samples. Black curve—density plot of the mean (panel “Mean”), minimum (panel “Min”) and maximum (panel “Max”) of the 5061 observed transcript abundances. Grey dashed curve—density plot of the mean (panel “Mean”), minimum (panel “Min”) and maximum (panel “Max”) of the 5061 simulated transcript abundances

Figure 4 compares the mean, minimum and maximum of the 5061 observed transcript abundances from the 1043 patients in the TRAC study to the mean, min and max of the 5061 transcript abundances simulated from a multivariate normal distribution for 1043 participants/samples (Additional file 1). The density plot of the means (panel “Mean”), minimums (panel “Min”) and maximums (panel “Max”) of the simulated transcript abundances overlap with the density plot of the means, minimums and maximums of the observed transcript abundances, indicating that the simulated transcript abundances resemble the observed transcript abundances.

### Outcome simulation

The outcome in the mock prediction modelling study is whether a participant had a slow or fast clearing *P. falciparum* infection. Slow clearing is defined as a parasite clearance half-life ($P{C}_{1/2}$) > 5 h; fast clearing is a $P{C}_{1/2}$ $\le$ 5 h. In the TRAC study, 29% (299/1043) of participants had slow clearing infections, 70% (735/1043) had fast clearing infections, and for 1% (9/1043) the $P{C}_{1/2}$ could not be calculated. Typically, in sample size calculations we want to assume some of the predictors are associated with the outcome. To incorporate some association between the simulated microarray data (see section “Microarray data simulation”) and simulated outcome (slow/fast clearing infection), five genes were randomly selected to have transcript abundances associated with the outcome. A logistic regression model was used to simulate the binary outcome (slow/fast clearing infection) based on the five selected simulated predictors (further details provided in Additional file 1). The remaining 5056 transcript abundances are considered noise or not associated. 1043 outcome values were simulated and the percentage of slow clearing infections simulated was 27% (282/1043), which is similar to that observed in the TRAC study.

Conclusions regarding the sample size of omics studies of artemisinin resistant parasites should not be made based on the results of this mock study/dataset. While the simulated transcriptomics data resembles the observed transcriptomics data (Fig. 4), the simulated outcome is generated from five randomly selected (simulated) transcripts and the magnitude of the association between these transcripts and the outcome was selected to generate an outcome proportion similar to that in the observed dataset (i.e., does not reflect the actual relationship between these transcripts and slow/fast clearing infections). The purpose of the simulated data is to illustrate how to derive learning curves for data of this nature and to interpret the curves.

### Statistical methods

Learning curves were constructed using two approaches. The first was a single train/test split approach where the simulated data (based on TRAC data) were randomly split into training and test datasets using an 80:20 ratio. This results in 835 individuals in the training dataset and 208 individuals in the test dataset. The training dataset of 835 individuals was then split into six smaller training datasets by randomly sampling without replacement 20, 40, 80, 160, 320, 640, and 835 individuals from the 835 individuals. The prediction models (described next in section “Prediction models”) were trained on each of the seven training sets. The trained model for each training dataset was used to make predictions on the test dataset of 208 individuals, which stays the same size across the seven training datasets. The seven training dataset BERs and corresponding seven test dataset BERs were both plotted against training dataset size to generate the learning curves for each prediction model.

The second approach is K-fold cross-validation. K-fold cross-validation generates K estimates of a model’s prediction error, that are averaged, to derive a more accurate estimate of a model’s prediction error on unseen data. This approach involves randomly dividing the dataset (all 1043 individuals) into K groups, or folds, of roughly equal size [16, 18]. K = 5 was selected for the mock study, resulting in 5 folds of size 208 or 209. For each training dataset size, the following was performed:

1. One of the 5 folds was selected as the test dataset (either 208 or 209 individuals), and the remaining data of either 835 or 834 individuals was used as the training dataset. For the smaller training dataset sizes (n = 20, 40, 80, 160, 320 or 640), n individuals were sampled without replacement from the training dataset of 835 or 834 individuals.
2. The BER is then computed on the training dataset and the test dataset.
3. This procedure is repeated 5 times; each time, a different fold is treated as a test dataset.
4. This process results in 5 estimates of the training BER and test BER, as opposed to the first approach, which produces a single estimate of the training BER and test BER for each training dataset size.

For each training dataset, the average of the training BERs and test BERs for a particular training dataset size, along with the min and max BER values, were plotted against training dataset size to generate the learning curves for each prediction model. For the last/largest training dataset size (835), the training BERs and test BERs are averaged across all 5 folds regardless of whether there were 835 or 834 in the training dataset or 208 or 209 in the test dataset.

### Prediction models

Learning curves were constructed for two prediction models developed using machine learning algorithms. Note, throughout this section the training dataset refers to one of the seven training datasets of different sample sizes described in the previous section. The first machine learning method was Breiman's random forest algorithm [21] implemented using the defaults of the randomForest function from R’s randomForest package [22].

The second was sparse Partial Least Squares-Discriminant Analysis plus Support Vector Machines (sPLSDA + SVM). In this combined approach, sPLSDA projects the predictors onto a domain of latent variables which are a linear combination of the predictors. By selecting a subset of the latent variables that best discriminate between the outcome (slow/fast parasite clearance), the number of predictors can be reduced, e.g., from 5061 predictors to 5 latent variables. SVM classifies observations by deriving non-linear boundaries (of dimension equal to the number of predictors) that separate the samples/individuals within each outcome category. The latent variables derived by sPLSDA were then used as predictors in SVM.

sPLSDA + SVM was implemented using splsda from R’s mixOmics package [23] and svm from R’s e1071 package [24, 25]. Further details on the prediction models are provided in Additional file 1.

All analyses were implemented in R version 4.4.1 [26]. The simulated data and R code to produce the learning curves presented in the Results section are available from: https://gitlab.unimelb.edu.au/sophiez/learning-curves-tutorial.

## Results

**Fig. 5:**
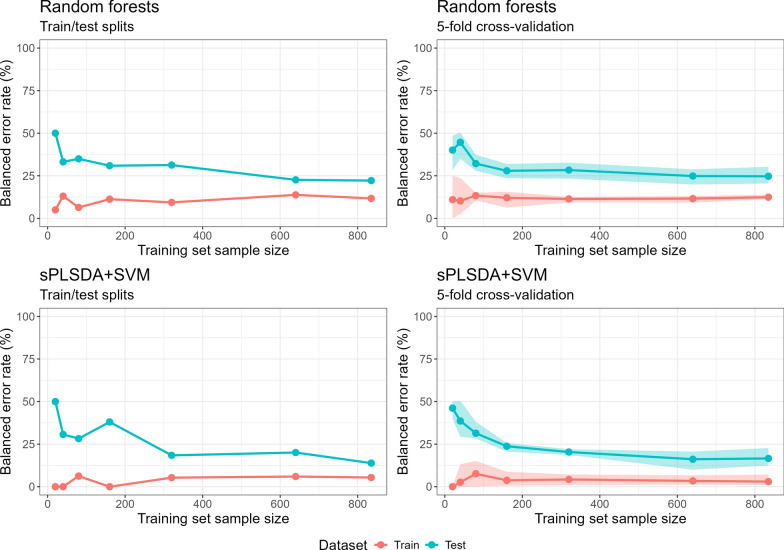
Learning curves produced by a single train/test split (left column) and fivefold cross-validation (right column) for machine learning prediction models using random forests (top row) and sPLSDA + SVM (bottom row). The predictors in the training and test sets were simulated transcript abundances for 5061 *P. falciparum* genes and the outcome was a simulated binary outcome indicating whether a participant has a fast or slow clearing *P. falciparum* infection. The size (number of samples/individuals) in the training set is indicated on the x-axis. The size of test dataset is 208 for train/test split and can vary between 208 and 209 for fivefold cross-validation (see “Statistical Methods” for further details). Shaded regions are the min and max balanced error rates observed for the 5 training dataset folds and 5 test set folds at each training dataset size. Balanced error rate is the average of the percentage of slow clearing infections incorrectly predicted as fast and percentage of fast clearing infections predicted as slow

The learning curves produced by a single split and fivefold cross-validation for both prediction models are presented in Fig. 5. The BERs plotted in Fig. 5 are provided in Table 2. For each training dataset (training BERs), the percentage of (simulated) participants incorrectly classified within each outcome category as either slow or fast clearing parasites, on average, was lower for sPLSDA + SVM than for random forests (Fig. 5 and Table 2).

**Table 2:** Balanced error rates (as plotted in Fig. 5)

	Random Forests		sPLSDA + SVM	
Training dataset size	Train/test split	fivefold cross-validation	Train/test split	fivefold cross-validation
	Training dataset balanced error rate (%)			
20	5.0	11.0 [0.0, 25.0]	0.0	0.0 [0.0, 0.0]
40	13.0	10.2 [2.5, 23.7]	0.0	2.6 [0.0, 13.2]
80	6.4	13.4 [10.5, 15.0]	6.2	7.6 [0.0, 15.1]
160	11.2	12.1 [6.3, 15.6]	0.0	3.8 [0.6, 8.8]
320	9.4	11.4 [9.4, 12.6]	5.3	4.3 [0.9, 7.2]
640	13.8	11.6 [9.2, 13.1]	6.0	3.4 [1.3, 6.7]
835	11.7	12.4 [11.3, 13.9]	5.4	3.0 [1.1, 7.1]
Test dataset balanced error rate (%)				
20	50.0	40.1 [28.3, 48.6]	50.0	46.2 [40.0, 50.0]
40	33.2	44.7 [35.0, 50.3]	30.7	38.6 [29.3, 50.3]
80	35.0	32.2 [28.0, 37.3]	28.2	31.4 [28.4, 38.2]
160	30.9	27.9 [23.6, 32.0]	38.0	23.8 [20.6, 25.7]
320	31.3	28.4 [23.3, 32.7]	18.4	20.4 [18.7, 22.0]
640	22.6	24.9 [19.8, 28.8]	20.1	16.1 [10.0, 20.6]
835	22.2	24.7 [20.5, 30.3]	13.9	16.6 [12.4, 22.7]

^*^Size of test dataset is 208 for train/test split. Can vary between 208 and 209 for fivefold cross-validation (see “Statistical Methods” for further details)

Balanced error rates for single train/test splits and average [minimum, maximum] balance error rates for fivefold cross-validation are presented for each machine learning prediction model (random forests and sPLSDA + SVM). The predictors in the training and test sets were simulated transcript abundances for 5061 *P. falciparum* genes and the outcome was a simulated binary outcome indicating whether a participant has a fast or slow clearing *P. falciparum* infection. The size (number of samples/individuals) in the training set is indicated on the x-axis. The size of test dataset is 208 for train/test split and can vary between 208 and 209 for fivefold cross-validation (see “Statistical Methods” for further details)

The percentage of participants incorrectly classified within each outcome category as either slow or fast clearing, on average, for the test dataset (test BERs) tended to be higher for random forests compared to sPLSDA + SVM: single split BERs range from 50.0 to 22.2% (as training dataset size increases) and K-fold cross-validation BERs from 40.1% [min, max: 28.3, 48.6] to 24.7% [20.5, 30.3] for random forests; and single split BERs range from 50.0% to 13.9% and fivefold cross-validation BERs random from 46.2% [40.0, 50.0] to 16.6% [12.4, 22.7] for sPLSDA + SVM.

For both machine learning models, the training and test curves have converged towards low BERs. In this scenario increasing the training dataset sizes beyond 835 is unlikely to reduce the percentage of participants incorrectly classified within each outcome category as either slow or fast clearing for the test dataset (test BERs), on average, to values much lower than those achieved at the training dataset size of 835 (single split BER 22.2% and fivefold cross-validation BER 24.7% [20.5, 30.3] for random forests; single split BER 13.9% and fivefold cross-validation BER 16.6% [12.4, 22.7] for sPLSDA + SVM).

A possible sample size statement/justification for a study protocol/journal article/grant based on the learning curves constructed using K-fold cross-validation (for example) could be:

Learning curves for prediction models developed using random forests and sPLSDA+SVM were constructed using 5-fold cross-validation from simulated outcome (slow clearing (parasite clearance half-life (PC1/2) > 5 hours) *P. falciparum* infection) and predictor (*in vivo* transcriptomics measurements) data. For a sample size of 1043, 5061 *in vivo* transcriptomics measurements were simulated from a multivariate normal distribution with mean vector and covariate matrix set to the sample means and sample covariance matrix of *in vivo* transcriptomics measurements from a previously published study (1043 samples, 5061 transcriptomics measurements per sample) [19]. Five of the simulated transcriptomics measurements were randomly selected to be associated with the simulated outcome, which was simulated from a logistic regression model. 27% (282/1043) of the simulated outcomes were slow clearing *P. falciparum* infections.

The learning curves indicate that both prediction models have converged towards a low percentage of participants incorrectly classified within each outcome category as either slow or fast clearing, on average, (balanced error rate (BER)) at the maximum training dataset size explored (835 samples). sPLSDA+SVM appears to be converging to a lower BER than random forests and hence will be selected as the machine learning method for the prediction modelling of the proposed study. A minimum sample size of 835 malaria patients is required to develop a prediction model using sPLSDA+SVM that can achieve a balanced error rate of 16.6% [min, max: 12.4, 22.7] for the prediction of slow clearing *P. falciparum* infections using *in vivo* transcriptomics data.

## Discussion

With the increasing use of ML methods in malaria research (e.g., to predict drug resistance/vaccine protection using omics and antibody data), it is important to consider a priori how sensitive model predictions are to training dataset size to ensure predictions for new individuals in the target population are reliable. As demonstrated by this tutorial, learning curves are a straightforward approach to sample size calculations for complex prediction models developed using machine learning techniques and have the potential to strengthen the sample size justification in grant applications and study protocols.

The advantages of the learning curves approach are that it is not specific to a particular type of machine learning method (e.g. many malaria prediction model papers have used multiple machine learning methods for robustness of findings), study design (cross-sectional, longitudinal) or outcome scale (continuous, binary/categorical). Using K-fold cross-validation to generate learning curves can also indicate whether including different samples/observations in a training set of a particular size leads to greatly different prediction model accuracy/performance (e.g., see the shaded regions in Fig. 5).

The main limitation of the approach is that it requires data from a previous study or simulated data. In this tutorial, falciparum malaria transcriptomics (predictor) data were simulated using the sample correlation matrix from a previous study [19]. A statistical model was used to simulate whether a participant had a slow or fast clearing falciparum malaria infection ($P{C}_{1/2}$> 5 h) based on the simulated transcriptomics data. The outcome simulation model was adjusted to simulate a proportion of slow clearing infections similar to that observed in a previous study [20]. Alternatively, functions/programmes to simulate omics data are becoming available in statistical software, such as the R packages OmicsSIMLA [27], MOPower (mainly focused on sample size for association studies, but does generate simulated omics data) [28] and MOSim [29]. An increasing amount of omics data is becoming publicly available each year in databases, such as OmicsDI [30] (can search databases by organism) and could be used to generate learning curves to inform the sample size of future malaria studies. An extension of learning curves where a curve is fitted to the learning curve and then the model performance at larger sample sizes is extrapolated from the fitted curve, could be used to explore larger sample sizes than those achieved in previous studies [12, 31].

Another limitation is that depending on the complexity of the prediction model development process, learning curves may need to be implemented with user written code. The caret package in R [32] and the scikit-learn library in Python [33] could be explored to simplify the programming involved to produce learning curves.

As with any sample size calculation, the accuracy of the sample size determined from learning curves depends on the quality and representativeness of the data (observed or simulated) being used [10].

## Conclusions

Learning curves are a simple and model agnostic approach that can be used to determine the minimum sample size required for future prediction modelling studies of different malaria outcomes that use machine learning algorithms for prediction.

## Supplementary Information

Additional file 1.

## Data Availability

The simulated dataset used in this tutorial was based on the transcriptomics dataset published in Mok et al. 2015 [19] (publicly available from: https://www.ncbi.nlm.nih.gov/geo/query/acc.cgi?acc = GSE59099) and the parasite clearance half-lives provided in the Supplementary Material of Mok et al. 2015 [19]. The simulated data and R code used to generate the learning curves in Fig. 5 are available from: https://gitlab.unimelb.edu.au/sophiez/learning-curves-tutorial.
