# Supplementary material for: How to use learning curves to evaluate the sample size for malaria prediction models developed using machine learning algorithms
Source: Malar J. 2025 Jul 24;24:242. doi: 10.1186/s12936-025-05479-3 (PMC12291394; doi:10.1186/s12936-025-05479-3)
Supplement: Supplementary file 1 — Additional file 1. [file 12936_2025_5479_MOESM1_ESM.docx]

**Additional file 1**

**How to use learning curves to evaluate the sample size for malaria prediction models developed using machine learning algorithms**

**Methods**

**Microarray data simulation**

Microarray data were simulated based on the Tracking Resistance to Artemisinin Collaboration (TRAC) study transcriptomics data published in Mok et al. 2015 [1].

2229 out of the 5061 *P. falciparum* genes had no missing transcript levels. For those genes with missing transcript levels (remaining 2832), the median number of missing values was 7 ((interquartile range: 2, 25) [min, max: 1, 338]). The missing observations in the TRAC transcriptomics data were replaced with the mean of the non-missing observations for that variable (i.e., mean imputed). The 5061 $\times$ 5061 sample covariance matrix derived from the transcriptomics data (observed data plus mean imputed values) was not positive definite, consequently singular value decomposition (SVD) was used to compute the square root of the sample covariance matrix. A total of 1043 vectors (i.e. 1043 individuals in the TRAC dataset) of length 5061 (i.e. 5061 transcriptomes per individual) were simulated from the following affine transformation of a standard multivariate normal distribution:

| $y=\mu+U\Lambda^{1/2}z$ | (S1) |
| --- | --- |

In equation (S1), $\mu$ is a $P$ (=5061) $\times$ 1 vector containing the sample means of the non-missing transcriptomics measurements (not mean imputed); $U$ and $\Lambda$ are derived from the SVD of the sample covariance matrix resulting from the observed plus mean imputed transcriptomics data. $\Lambda^{1/2}$ = $\text{diag}\left( \lambda_{1}^{1/2},\ldots,\lambda_{P}^{1/2} \right)$ is a $P$ $\times$ $P$ diagonal matrix containing the square root of the singular values ($\lambda_{p}$ for $p = 1, \ldots, P$) from the SVD arranged in descending order and $U$ = $\left[ u_{1},\ldots,u_{P} \right]$ is an orthogonal matrix where each column $u_{p}$ is the $P$ $\times$ 1 left singular vector that corresponds to the singular value $\lambda_{p}$. Let $M<P$ be the sum of the nonzero singular values or the rank of the sample covariance matrix. $z$ is a $M$ $\times$ $N$ (=1043, i.e., the number of samples) matrix containing values sampled from a standard normal distribution. $z$ is extended with $P-M$ rows of zeros to make it a $P$ $\times$ $N$ matrix, and then equation (S1) is evaluated to generate $y$, a $P$ $\times$ $N$ matrix containing the simulated transcriptomics data.

**Outcome simulation**

The outcome to be predicted in the mock prediction modelling study is whether a participant had a slow or fast clearing *Plasmodium falciparum* (Pf) infection. Slow clearing is defined as a parasite clearance half-life ($PC_{1/2}$ > 5 hours); fast clearing is a $PC_{1/2}$ $\leq$ 5 hours. In the TRAC study, 29% (299/1043) of participants had slow clearing infections, 70% (735/1043) had fast clearing infections, and for 1% (9/1043) the $PC_{1/2}$ could not be calculated (in Supplementary Material of Mok et al. 2015 [1]). The following was performed to simulate a percentage of slow clearing infections similar to that observed in the TRAC study. Typically, in sample size calculations we want to assume some of the predictors are associated with the outcome. To incorporate some association between the simulated microarray data and simulated outcome (slow/fast clearing infection), five genes were randomly selected to have transcript abundances associated with the outcome (see Table S1 for the randomly selected genes). The outcome was then simulated from the following logistic regression model:

| $y_{i}\sim\text{Bern}\left( p_{i} \right)$ | (S2) |
| --- | --- |
| $\text{logit}\left( p_{i} \right)=\beta_{0}+\beta_{1}x_{1i}+\beta_{2}x_{2i}+\beta_{3}x_{3i}+\beta_{4}x_{4i}+\beta_{5}x_{5i}$ |  |

In equation (S2), $y_{i}$ is a binary outcome variable that takes value 1 for a slow clearing infection and 0 for a fast clearing infection and is assumed to follow a Bernoulli distribution (Bern); $p_{i}=Pr\left( y_{i}=1 \right)$ is the probability of a slow clearing infection$; x_{1i}$ to $x_{5i}$ represent the simulated transcript levels of the genes in Table S1; the remaining 5056 transcript levels are considered noise or not associated; $\beta=\left( \beta_{0},\ldots,\beta_{5} \right)$ are regression coefficients; $\beta_{0}$ is the intercept term and represents the log-odds of a slow clearing infection when the transcript levels (predictors) are 0; $e^{\beta_{0}}$ is the odds of a slow clearing infection when the transcript levels are 0; $\beta_{j}$, for $j=1, \ldots, 5$, is the change in log-odds of a slow clearing infection for a unit increase in simulated transcript levels, after holding the other transcript levels constant; $e^{\beta_{j}}$ the fold-change in odds of a slow clearing infection for a unit increase in simulated transcript levels, after holding the other transcript levels constant. The regression coefficients ($\beta$) in equation (S2) were set to the values in Table S1 and predicted probabilities for all 1043 samples calculated. Predicted probabilities greater than 0.5 were considered slow clearing infections. 1043 outcome values were simulated and the percentage of slow clearing infections simulated by equation (S2) was 27% (282/1043), which is similar to that observed in the TRAC study.

Table S1: Regression coefficient values used to simulate a binary outcome variable (slow/fast clearing infection) from equation (S2). The genes randomly selected to have simulated transcript abundances associated with the outcome are listed in the Term column.

| Term | Covariate^a^ | $\beta$^a^ | Value^b^ | Exp(value)^c^ |
| --- | --- | --- | --- | --- |
| Intercept | N/A | $\beta_{0}$ | -0.50 | 0.61 |
| PF3D7_0406100 | $x_{1}$ | $\beta_{1}$ | 0.30 | 1.35 |
| PF3D7_0501500 | $x_{2}$ | $\beta_{2}$ | -0.15 | 0.86 |
| PF3D7_1002200 | $x_{3}$ | $\beta_{3}$ | 0.35 | 1.42 |
| PF3D7_1406100 | $x_{4}$ | $\beta_{4}$ | 0.40 | 1.49 |
| PF3D7_0416800 | $x_{5}$ | $\beta_{5}$ | -0.15 | 0.86 |

N/A – not applicable

^a^Covariate symbol in equation (S2) and corresponding logistic regression coefficient ($\beta$) symbol in equation (S2).

^b^Simulated outcome was generate by setting the regression coefficients in equation (S2) to these values. Represent the log-odds of a slow clearing infection when transcript levels are 0 ($\beta_{0}$) or change in log-odds of a slow clearing infection for a unit increase in transcript levels, after holding the remaining transcript levels constant ($\beta_{j}$, for $j=1, \ldots, 5$). The beta values were selected to simulate a proportion of slow/fast clearing infections close to that in the observed dataset and do not reflect the actual magnitude of the associations between these variables and the outcome in the real dataset.^C^Corresponding odds of a slow clearing infection when the transcript levels are 0 ($e^{\beta_{0}}$) or fold-change in odds of a slow clearing infection for a unit increase in simulated transcript levels, after holding the other transcript levels constant ($e^{\beta_{j}}$).

**Prediction models**

The tune.splda function from the mixOmics package was used to select the optimal number of latent variables from a maximum of 5 and to determine the number of predictors used to construct each latent variable from the following user specified values: 1, 2, 3, 4, 5, 6, 7, 8, 9, 10, 20, 30, 50, 70 and 100. In the tune.splda function 5-fold cross-validation repeated 5 times was specified and the optimal number of latent variables and predictors to include on each latent variable that minimised the BER was selected. The BER was calculated using classifications based on the Mahalanobis distance. sPLSDA was then performed on the full training dataset (of a particular size) with these optimal values. SVM was then performed on the latent variables derived from the trained sPLSDA model using C-classification, cost value of 0.8, and a radial kernel with gamma parameter set to 0.7.

Synthetic Minority Oversampling Technique (SMOTE) [2] implemented using the SmoteClassif function in R’s UBL package [3] was used to handle imbalanced classes in the training datasets. The SMOTE method produces balanced classes by generating new examples of the minority (lowest frequency) class using the k (equal to the default of 5) nearest neighbors algorithm, as well as under-sampling the majority (highest frequency) class. The Euclidean (default) distance metric was specified for the k nearest neighbor algorithm. The TomekClassif function in R’s UBL package was then applied to the SMOTE’d training dataset to remove samples belonging to different classes that happened to be very close to each other [4].

The trained random forest and sPLSDA+svm algorithms were then used to generate the predicted probability of a slow clearing infection for each sample in both the training dataset and test dataset. The BER was derived by determining a single cutpoint that maximized the Youden-Index on the training dataset using the cutpointr function from R’s cutpointr package [5]. Predicted probabilities derived from the train/test datasets that were greater than or equal to this cutpoint were classified as slow clearing.

**References**

1. Mok S, Ashley EA, Ferreira PE, Zhu L, Lin Z, Yeo T, et al. Population transcriptomics of human malaria parasites reveals the mechanism of artemisinin resistance. Science. 2015;347:431–5.

2. Chawla NV, Bowyer KW, Hall LO, Kegelmeyer WP. SMOTE: Synthetic Minority Over-sampling Technique. Journal of Artificial Intelligence Research. 2002;16:321–57.

3. Branco P, Ribeiro RP, Torgo L. UBL: an R package for Utility-based Learning [Internet]. arXiv; 2016 [cited 2024 Jul 7]. Available from: http://arxiv.org/abs/1604.08079

4. Batista GEAPA, Prati RC, Monard MC. A study of the behavior of several methods for balancing machine learning training data. SIGKDD Explor Newsl. 2004;6:20–9.

5. Thiele C, Hirschfeld G. cutpointr: Improved Estimation and Validation of Optimal Cutpoints in R. Journal of Statistical Software. 2021;98:1–27.
